# Ultrahigh numerical aperture meta-fibre for flexible optical trapping

**DOI:** 10.1038/s41377-021-00491-z

**Published:** 2021-03-15

**Authors:** Malte Plidschun, Haoran Ren, Jisoo Kim, Ronny Förster, Stefan A. Maier, Markus A. Schmidt

**Affiliations:** 1 Leibniz Institute of Photonic Technology, 07745 Jena, Germany; 2 Abbe Center of Photonics and Faculty of Physics, FSU Jena, 07745 Jena, Germany; 3 Chair in Hybrid Nanosystems, Nanoinstitute Munich, LMU München, 80539 München, Germany; 4 Department of Physics, Imperial College London, London, SW7 2AZ UK; 5 Otto Schott Institute of Material Research, FSU Jena, 07745 Jena, Germany

**Keywords:** Fibre optics and optical communications, Optical manipulation and tweezers, Transformation optics, Biophotonics

## Abstract

Strong focusing on diffraction-limited spots is essential for many photonic applications and is particularly relevant for optical trapping; however, all currently used approaches fail to simultaneously provide flexible transportation of light, straightforward implementation, compatibility with waveguide circuitry, and strong focusing. Here, we demonstrate the design and 3D nanoprinting of an ultrahigh numerical aperture meta-fibre for highly flexible optical trapping. Taking into account the peculiarities of the fibre environment, we implemented an ultrathin meta-lens on the facet of a modified single-mode optical fibre via direct laser writing, leading to a diffraction-limited focal spot with a record-high numerical aperture of up to NA ≈ 0.9. The unique capabilities of this flexible, cost-effective, bio- and fibre-circuitry-compatible meta-fibre device were demonstrated by optically trapping microbeads and bacteria for the first time with only one single-mode fibre in combination with diffractive optics. Our study highlights the relevance of the unexplored but exciting field of meta-fibre optics to a multitude of fields, such as bioanalytics, quantum technology and life sciences.

## Introduction

Strong focusing of light onto diffraction-limited spots is one of the fundamental prerequisites for a vast number of optical applications, including high-resolution imaging, microscopy, optical manipulation, and material processing through optical lithography. In particular, precise optical control and manipulation of micro- and nanoscopic objects by focused laser beams^1^ have led to sophisticated applications in biophotonics^2–5^, microfluidics^6–8^, and quantum technology^9,10^. Here, stable and robust optical trapping demands a strong gradient force along the three spatial directions to provide a strong trapping force in the radial direction and to counterbalance the scattering force along the axial direction. As such, tight light focusing is conventionally achieved by using microscope objectives with a very high numerical aperture (NA > 0.8); however, these optical components are bulky and suffer from a lack of flexibility and integrability, poor remote operating capabilities, and high cost and demand for large-volume infrastructure.

Optical fibres open up the possibility to address the abovementioned limitations and represent a flexible, remotely operable, and low-cost photonic platform. In particular, the use of single-mode fibres (SMFs) offers a major advantage for producing output beams with well-defined optical properties. Unlike multimode fibres (MMFs) that include many modes, SMFs support only the low-loss fundamental mode. This gives rise to a well-controlled output beam that is insensitive to external influences and yields a uniform phase profile, which is essential for wavefront manipulation. Moreover, SMFs allow remote distances to be reached when light propagation is unaffected by the length of the optical fibre, while MMFs are highly susceptible to fibre bending. Nevertheless, the use of optical fibres in focusing applications and particular optical trapping is fundamentally limited by the divergence of light emerging from the fibre facet. With respect to optical trapping, several approaches have been proposed to overcome divergence, including dual-fibre arrangements with counterpropagating beams^4,11–13^ and MMFs in combination with an iterative adaptive compensation technique^14^. Although trapping has been successfully demonstrated, the associated systems include complicated and cumbersome optical components that are not suitable for in vivo applications.

Recent advances in nanotechnology provide unprecedented opportunities to shape optical beams via the use of ultrathin meta-surfaces that are composed of subwavelength elements, and, thus, allow digitization and tailoring of wavefronts at interfaces^15,16^. In this context, implementation of meta-lenses on fibre tips for tight light focusing has been realized via, e.g., focused ion-beam milling^17,18^ and chemical etching^19^; however, the realized meta-lenses only reach a maximum NA of 0.37 (ref. ^18^). On the other hand, three-dimensional (3D) direct laser writing (DLW) based on two-photon polymerization, offers an ideal platform to optically digitalize arbitrary 3D nanostructures on substrates that are difficult to handle otherwise^20,21^. Although first attempts have recently been made to optically digitize diffractive Fresnel lenses on a fibre tip^22^, a dual fibre arrangement for optical trapping is still required due to the limited maximum NA of 0.7. Therefore, implementing an ultrahigh NA meta (UNM)-lens on a fibre tip that allows for tight focusing of light in an in vivo environment remains an open challenge, while such an arrangement represents a powerful approach for a multitude of applications, examples of which include flexible optical trapping and high-resolution scanning microscopy.

**Fig. 1:**
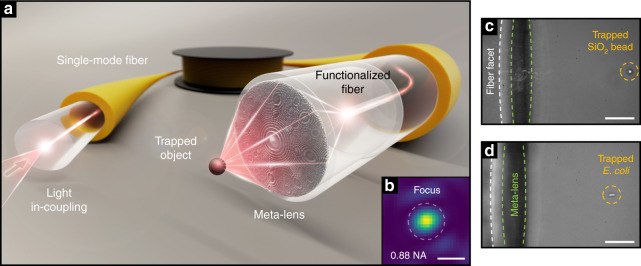
Principle of an ultra-high NA meta-fibre for flexible optical trapping. **a** Illustration of the device including a 3D nanoprinted meta-lens located on the tip of an optical fibre to trap particles. **b** Example of a measured radial focal plane in water (scale bar: 500 nm, wavelength of 660 nm, the dashed line indicates the width of the fitted Airy function). **c** Micrograph of a 2 µm silica bead and (**d**) an *Escherichia coli* (*E. coli*) bacterium optically trapped with a single fibre UNM in water imaged from the side (scale bars 15 µm)

**Fig. 2:**
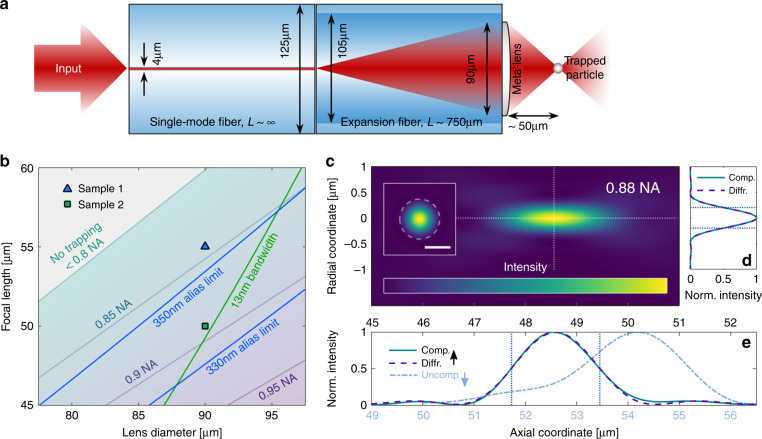
Design strategy and simulated focusing performance of the on-fibre UNM. **a** Detailed view of the functionalized fibre consisting of a single-mode fibre, spliced multimode fibre section for beam expansion, and the meta-lens on its facet that is used to create a tight focus for trapping particles. **b** Meta-lens design map: Limiting factors for UNM design (*λ*_0_ = 660 nm). Low spatial resolution results in aliasing, while limited laser source bandwidth leads to incoherent interference of the Fresnel zones. **c** Simulated focal spot of an on-fibre meta-lens including wavefront curvature correction. The inset shows the radial focal plane (scale bar: 500 nm, dashed line: with the fitted Airy function; note that the scale of both axes is the same as that in the inset and in (**e**)). The colour scale ranges linearly from zero (dark blue) to unity (yellow). **d** Radial and (**e**) axial (upper axis) intensity profiles (solid) along the symmetry axes and the corresponding fits (dashed). The vertical dotted lines indicate the full-width-half-maximum of the fits. Note that in (**e**), the uncompensated fibre wavefront curvature (blue, lower axis) leads to a strong focal shift and spherical aberration compared to the wavefront-corrected design (green, upper axis)

Here, we demonstrate the design and 3D nanoprinting of an UNM on the facet of a functionalized SMF, underpinning an UNM-fibre device for flexible optical trapping based on a single single-mode fibre (Fig. 1a). Specifically, we show the design and experimental realization of UNMs with spatial resolutions of down to 300 nm, taking into account the peculiarities of the fibre environment. By reaching record-high numerical apertures of up to ≈0.9 and diffraction-limited spots (Fig. 1b), we reveal the capabilities of our device by optically trapping microbeads and biologically relevant species for the first time with a single SMF in combination with diffractive optics. The implementation relies on 3D-DLW in combination with a functionalized SMF including a submillimetre piece of MMF for beam expansion to accommodate the full numerical aperture of the UNM (Fig. 2a). This allows us to expand the cross-section of the output beam of the SMF to a significantly larger lateral dimension (~95 µm in diameter), which would otherwise be too small (~4 µm in diameter) to enable sufficient wavefront manipulation. Flexible remote optical trapping of silica microbeads (Fig. 1c) and *Escherichia coli* (*E. coli*) bacteria (Fig. 1d) is experimentally demonstrated, confirming the unique focusing performance of our device (see Movies S1–S6).

## Results

### Design of an UNM-enhanced meta-fibre

Interfacing an UNM with an optical fibre requires careful geometric and phase design to include the specific circumstances of the fibre environment in terms of both beam properties and geometric constraints.

#### Meta-lens design

Experimental implementation of the UNM used here with a focal point at an axial distance *z* = *f* relies on 3D-DLW, requiring discretization of a hyperbolic phase $\phi _{{\mathrm{hyp}}}\left( {r,f} \right) = - 2\pi n/\lambda _0 \cdot \left( {\sqrt {r^2 + f^2} - f} \right)$ (*n*: refractive index of the medium, *λ*_0_: vacuum wavelength, *r*: radial coordinate). The phase profile *ϕ* is discretized in steps of 2*π* into Fresnel zones via *ϕ*_kin_ = mod(*ϕ*,2*π*), leading to a kinoform-type phase distribution^23–25^ (details in Supplementary Results 1). Note that the hyperbolic phase profile exhibits less critical curvature towards the edges than its parabolic counterpart, and is, thus, used throughout this work. In contrast to a multilevel diffractive Fresnel lens^23,24^ with locally adaptive sampling, the globally constant in-plane discretization Δ*x* establishes limits to the interpixel phase change that can be spatially resolved (|*ϕ*′| ≈ |Δ*ϕ*/Δ*x*| *<* *π*/Δ*x*), which is defined by the Nyquist–Shannon theorem^26^, and reduces the effectively usable lens diameter fraction and connected NA for the case of undersampling, i.e., aliasing. Here, we choose a globally constant spatial resolution of Δ*x* = 300 nm to ensure alias-free operation at λ_0_ = 660 nm (see Supplementary Results 2 and Fig. S1 for details), which results in a constant in-plane pitch for the elements (Fig. S6), similar to the arrangement of a metasurface. This is different from a multilevel diffractive Fresnel lens^23,24^, which relies on locally variable in-plane pitches that yield a limited NA when the spatial resolution of the implementation approach (in the present case of 3D nanoprinting technology) is on the order of hundreds of nanometres. To efficiently make use of the entire lens cross-section (i.e., NA, chosen here to be 2*r* = 90 µm), constructive interference of wavefronts emerging from all lens elements is required, which is limited by the coherence length of the laser source *L*_coh_ = *λ*_0_^2^/(*n*Δ*λ*_0_) (bandwidth Δ*λ*_0_). Note that the highest lens performance in terms of NA is achieved for the case when all Fresnel rings are taken into account, i.e., if *L*_coh_ > *λ*_0_*N*_Fres_ (*N*_Fres_: number of Fresnel rings, see details in Supplementary Results 3). All mentioned issues allow us to define a meta-lens design map (Fig. 2b) including the alias limit and bandwidth of the laser source used (Δ*λ*_0_ = 13 nm), showing that the two experimentally targeted configurations (sample 1: Δ*x*_1_ = 330 nm, *f*_1_ = 55 µm; sample 2: Δ*x*_2_ = 300 nm, *f*_2_ = 50 µm) are in line with the limits imposed by the nanoprinting process.

#### Fibre-wavefront compensation

Particular emphasis must be placed on the divergence of the beam emitted by the fibre to achieve maximum meta-lens performance. For conventional step-index fibres, wavefronts are curved with radius *R*(*z*) *=* *z* · (1 + *z*_R_^2^/*z*^2^) ≈ *z* for propagation distances significantly larger than the Rayleigh length (*z* ≫ *z*_R_ ∼ 10…30 µm in the visible spectrum). Hence, the fibre acts as a negative defocusing lens with *f* *=* −*R*(*z*) and exhibits its own spherical phase $\phi _{{\mathrm{fib}}}\left( {r,z} \right) = k \cdot \left( {\sqrt {r^2 + z^2} - z} \right)$, leading to an additional contribution to the overall phase *ϕ*_tot_(*r*,*f*,*z*) *=* *ϕ*_hyp_(*r*,*f*) *+* *ϕ*_fib_(*r*,*z*). It is straightforward to compensate for this spherical aberration (i.e., phase anomalies) by using DLW (see Fig. S2 for details), thus representing a key feature.

Figure 2c shows the simulated beam profile for a hyperbolic-phase meta-lens (sample 2, NA *=* 0.88), including the mentioned wavefront correction *ϕ*_tot_(*r*,*f*,*L*) − *ϕ*_fib_(*r*,*L*_min_ = 700 µm). Note that the output mode from the fibre freely expands in the MMF section across a length *L* ∼ 750 µm to fill the entire lens aperture. The use of an MMF is essential, as reflections and aberrations at the fibre junction are significantly reduced due to the matched refractive indices of both fibres, which is different from the case when a pure silica rod is used for beam expansion. Due to experimental tolerances in fibre cutting, a minimum length *L*_min_ = 700 µm for the expansion fibre is assumed in the meta-lens design, leading to an experimentally overcompensated fibre aberration, which, in general, has a less severe impact on lens performance than under-compensation. This is because a short section introduces stronger wavefront curvature than that possible for an overestimated design. Furthermore, the overestimation accounts for possible deviations of the actual wavefront from the theoretical fibre wavefront assumed in the design for the correct length. According to diffraction theory, the intensity profiles along the symmetry lines (Fig. 2d, e) were fitted along the radial *I*(*r*) ∼ jinc^2^(2π*r*/*λ*_0_·NA) and axial $I\left( z \right) \sim {\mathrm{sinc}}^2\left( {\left( {z - f} \right)/\lambda _0 \cdot \left( {n - \sqrt {n^2 - {\mathrm{NA}}^2} } \right)} \right)$ directions to retrieve the numerical aperture^27^ (see details in Supplementary Results 4). Note that an uncorrected phase profile reduces the NA by ~0.1 to *<*0.8, including a shift of Δ*f* ∼ 5 µm in the axial focal position. Overall, the results reveal that the specific design procedure outlined here allows for the implementation of meta-lens-enhanced fibre devices with unprecedented high numerical apertures of up to NA ≈ 0.9.

### Meta-fibre fabrication and characterization

#### Implementation

**Fig. 3:**
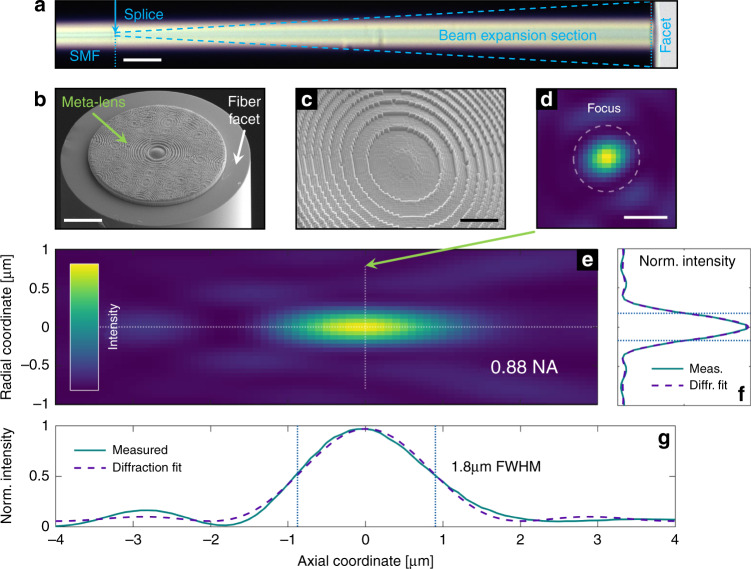
Measured focusing performance of the implemented UNM-enhanced meta-fibre (sample 2, *λ*_0_ = 660 nm in water). **a** Microscope image of a functionalized meta-fibre consisting of a single-mode fibre and spliced beam expansion section (scale bar 50 µm). **b**, **c** Scanning electron micrographs of an example implemented device (scale bars at 25 µm and 10 µm, respectively). **d** Measured cross-section of the beam taken at the focal plane (*z* = 0, scale bar: 500 nm). The dashed circle describes the width of a fitted Airy function. **e** Axial stack for measured beam cross-sections in cylindrical coordinates obtained by transforming each 2D image into radial symmetry via azimuthal averaging (note that the scale of both axes is the same as that in (**d**) and (**g**)). **f** Radial and (**g**) axial intensity profiles (solid) along the symmetry axes of the focus, with the dashed lines showing the corresponding fits. In all contour plots, the colour scale ranges linearly from zero (dark blue) to unity (yellow)

A 50 cm-long piece of conventional SMF (Thorlabs 630HP, mode diameter 4 µm, single-mode at *λ*_0_ = 660 nm) was fusion spliced to an MMF (Thorlabs FG105LVA, core diameter 105 µm) and used for beam expansion to exploit the full cross-section of the meta-lens (Fig. 3a). The spliced MMF is then cleaved to a length of approximately 700 µm (sample 1: *L*_1_ ∼ 720 µm, sample 2: *L*_2_ ∼ 750 µm), resulting in a beam (NA ≈ 0.09) spanning across the actual meta-lens aperture (2*r* = 90 µm, images of the fundamental mode output are shown in Fig. S5). UNM implementation includes a silanization step to improve adhesion (see details in “Materials and methods”) and 3D nanoprinting (Nanoscribe GmbH) using an IP-Dip photoresist. All implemented lenses have diameters of 2*r* = 90 µm, while two types of samples were produced (sample 1: Δ*x*_1_ = 330 nm, *f*_1_ = 55 µm (NA = 0.84); sample 2: Δ*x*_2_ = 300 nm, *f*_2_ = 50 µm (NA = 0.88), Fig. 3b, c). The final height of a meta-lens is *h* ∼ 3 µm, requiring an approximate printing time of 1 h (see Fig. S6 for close-up micrographs of the structure of a 3D nanoprinted UNM). The measured transmission through a UNM is >50% in the visible spectrum and significantly increases for longer wavelengths due to reduced scattering losses.

#### Performance of on-fibre UNM

The focusing performance of the meta-lens-enhanced fibre was determined by coupling light from a laser diode (Thorlabs L660P120, *λ*_0_ = 660 nm, bandwidth Δ*λ*_0_ = 13 nm) into the sample and immersing its tip into the water while measuring the transverse beam profile at different distances from the UNM surface (step size Δ*z* = 100 nm) with a high-NA water-dipping objective (Nikon 60×, NA = 1.0, see “Materials and methods” for further details). At each position, three micrographs with different exposure times were taken and combined to create a high-dynamic-range (HDR) image with a low noise floor of *<*10^−5^.

As an example, Fig. 3d–g shows the measured beam profile of sample 2, revealing extremely tight and diffraction-limited focusing (Fig. 3d) with the remarkable agreement with the beam profile of the designed UNM (Fig. 2c). The individual focal planes (see Fig. S7) are in excellent agreement with the azimuthal average presented in Fig. 3e and show no distortions into either of the directions. For a qualitative performance comparison, the measured intensity distributions along the symmetry axes of the focus (Fig. 3f: radial, Fig. 3g: axial) are fitted (dashed lines) using the same procedure applied for the meta-lens design, with the vertical dotted lines indicating the FWHM. The resulting values for the numerical aperture are extraordinarily high (NA ≈ 0.9) and match those obtained from simulations (Fig. 2c–e, see Supplementary Results 5 for a detailed analysis). Note that even a small spherical aberration at *z* = ±3 µm resulting from overcompensation of the wavefront curvature, i.e., due to the use of *L* ∼ 750 µm instead of 700 µm, is visible. Specifically, different slopes of the intensity decay before and after the focus are associated with positive spherical aberration and represent a remarkable feature in terms of the agreement between experiment and design.

### Meta-fibre-based flexible optical trapping

**Fig. 4:**
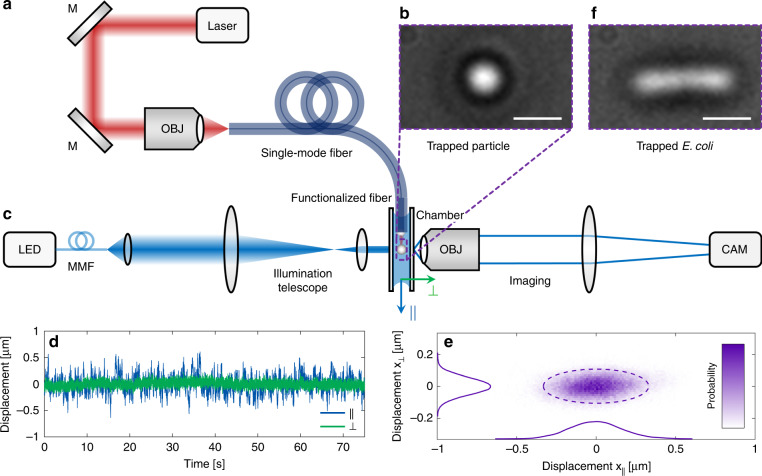
Optical trapping using a single UNM-enhanced SMF. **a** Sketch of the trapping setup consisting of a high-power laser (*λ*_0_ = 660 nm) coupled to an UNM-fibre sample, (**b**) windowed chamber with micro-objects immersed in water, and (**c**) Koehler illumination (left, a wavelength of 455 nm) and imaging system (right) used to record the motion of the trapped object. The top right images show micrograph examples of (**b**) a trapped 2 µm silica sphere and (**f**) a trapped *Escherichia coli* (*E. coli*) bacterium (scale bars: 2 µm). **d** Examples of spatial dynamic displacements for a trapped silica bead along with the transverse (green) and longitudinal (blue) directions for a trapping time of >1 min. **e** Corresponding 2D representation of the probability with projections on the respective axes. The dashed ellipse depicts the 2*σ*-environment containing 95% of the points

The unique properties of the meta-lens functionalized fibre device are demonstrated here by optically trapping freely diffusing micro-objects in water requiring at least NA > 0.8 (ref. ^28^). The trapping setup consists of a strong laser diode (Thorlabs L660P120, *λ*_0_ = 660 nm, coupled power at the sample output *P*_out_ = 37 mW, Fig. 4a), a windowed liquid chamber containing 2 µm silica beads dispersed in water (Fig. 4b) and the functionalized fibre sample (see details in “Materials and methods”). The motion of a trapped object is recorded from the side via a home-built microscope (sketch in Fig. 4c) with a fast frame rate (frequency 1 kHz) and very low exposure time (10 µs). The trajectory of a trapped bead (Fig. 4d) is determined from its position in each frame via a sophisticated data processing technique (see ref. ^13^ for details), yielding the dynamic displacement perpendicular (*x*_⊥_(*t*)) and parallel (*x*_||_ = *z*) to the fibre axis.

As for example shown for sample 2 (NA = 0.88), dielectric microbeads were successfully trapped over desirable timescales (here > 1 min, Fig. 4d) along with all three spatial directions within a region smaller than the area of the focus (Fig. 4e), demonstrating the capabilities of the UNM-enhanced fibre concept with respect to optical trapping (example of a trapped bead is shown in Fig. 4b). It should be mentioned that stable optical trapping was successfully achieved with all fabricated samples over the desired times, while the samples were continuously reused over the course of weeks with no sign of sample degradation observed, making the nanoprinted UNM approach a highly reproducible and reliable concept.

To demonstrate the capabilities of the meta-lens-enhanced fibre concept with respect to life science applications, a biologically relevant object (*E. coli* bacterium) was successfully trapped over the timescale of minutes and desirably released from the trap (Figs. 1d and 4f, also see Movies S5 and S6). No degradation of either bacterium or meta-lens was observed within subsequent trapping experiments for the same bacterium at powers of up to 37 mW in the focus. Note that the halo around the trapped objects results from the chosen low NA (0.16) of the Koehler illumination (details in “Materials and methods”). This ensures a great depth of field while maintaining sufficiently high contrast, facilitating image tracking (for details, see ref. ^13^) owing to larger coverage of the pixels.

To quantitatively assess the trapping performance of the meta-lens-enhanced fibre, we determined the trap stiffness at different power levels by analysing the dynamics of trapped beads. Specifically, power spectral density (PSD) evaluation^23^ and mean-square-displacement (MSD) analysis^29^ (both described in “Materials and methods”) were employed for determining the details for the spectral and temporal bead motion characteristics as well as for filtering out potential errors. A key benchmark performance parameter that allows for comparison to other trapping approaches is the optical trap stiffness *κ* = 2π*k*_B_*Tf*_c_/*D*, which characterizes the strength of the trap potential. This quantity is proportional to the ratio of the two key fit parameters, namely, corner frequency *f*_c_ ∼ 1/*τ*_c_ (*τ*_c_ time constant for confinement inside the trap) and free particle diffusion *D* (*k*_B_*T*: mean free thermal energy).

**Fig. 5:**
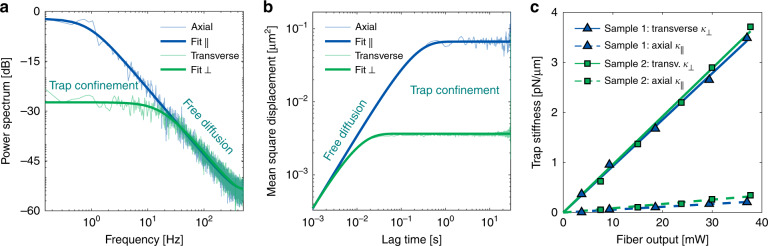
Analysis of the results from trapping silica beads using the UNM-enhanced fibre. **a** Power-spectral-density (PSD) analysis of a representative dataset (shown in Fig. 4d), including the corresponding fits (thick lines) for the axial (blue) and transverse (green) directions. **b** Corresponding mean-square-displacement (MSD) analysis for the same dataset including fits. **c** Resulting trap stiffness at different power levels for both samples (sample 1: NA = 0.84, sample 2: NA = 0.88, for details, see Fig. 2b) determined using the MSD method (Fig. 5b). As expected, a higher NA yields stronger trapping, while the transverse stiffness is approximately one order of magnitude higher than that in the axial direction (compare ref. ^13^)

The PSD for a measured trajectory (Fig. 4d) is presented in Fig. 5a and reveals two essential features: the plateau on the low-frequency side results from confinement inside the optical trap, while the linear slope at high frequencies is associated with free particle diffusion. Little to no aliasing from high-frequency noise due to motion blur is visible, resulting from a low exposure time of only 10 µs. Furthermore, no superimposed secondary oscillatory motion of the trapped bead appearing as a peak in the spectrum is present. In comparison, Fig. 5b illustrates the corresponding MSD/lag time dependence, which also shows a plateau for long lag times and a linear slope for short lag times, again describing confinement and free diffusion, respectively. Similar to the PSD analysis, no particle drift on time scales of >1 min is visible.

Overall, good agreement between MSD and PSD analysis for the retrieved fit parameters (*f*_c_ and *D*) was achieved, and, thus, we solely discuss the MSD results in the following. The final trap stiffness for the two implemented samples (sample 1: NA = 0.84, sample 2: NA = 0.88) was determined at different power levels for the transverse and axial directions independently (*κ*_⊥_ and *κ*_||_) and is shown in Fig. 5c. The transverse stiffness *κ*_⊥_ is approximately one order of magnitude larger than *κ*_||_ due to the elongated shape of the focus (ratio > 4:1), which directly impacts the distribution of the particle displacement (compare Figs. 3e and 4e). A comparison of the two implemented samples shows that sample 2 with NA = 0.88 exhibits a better trapping performance than sample 1 (NA = 0.84), i.e., yields stronger trapping due to tighter focusing. Note that such quantitative analysis of the trajectory of the trapped *E. coli* was not conducted here owing to the additional presence of rotational diffusion, demanding a more complex tracking routine.

### Comparison to other work

Considering previous work on meta-lens-enhanced fibres, it is worth mentioning that none of these approaches allows for optical trapping with a single fibre due to a low NA (Table 1). These methods demand the use of additional surfaces to overcome the axial scattering force^17,18,22^, preventing their use for in vivo applications. Our UNM-enhanced fibre resolves this issue and demonstrates full 3D optical trapping with a single device. It exhibits an extraordinarily high numerical aperture, which we believe is the highest confirmed value for a meta-lens functionalized optical fibre to date allowing for optical trapping, as demonstrated here for dielectric microparticles and *E. coli* bacteria. Other approaches that principally reach trapping-relevant numerical apertures (Table 1) require sophisticated and noncommercially available fibres and extensive additional equipment (e.g., spatial light modulators), all of which are not required here. In contrast, the presented approach solely includes commercial SMFs and MMFs, yielding significant advantages such as low cost, fibre length independence, compatibility with fibre circuitry and robustness against external influences such as bending, all of which are limiting factors in the previous work^14^. Another key benefit is the straightforward implementation procedure based on nanoprinting and splicing, avoiding the employment of clean-room-type machinery, including mask exposure, multistep processing, and large-scale and cost-intensive equipment^18,19,30–32^. An additional remarkable feature is the high damage threshold of our device, which overall results from an extended beam cross-section at the meta-lens location, effectively reducing the local intensity to a maximum value of *I*_peak_ ∼ 6 mW/µm^2^, which we found to be below the damage threshold. Note that stable trapping with each implemented sample was achieved over many trapping experiments (each of which lasted for minutes), and no sample degradation was observed over the course of weeks.

**Table 1:** Summary of the previously reported work

Working principle	Fabrication method	Lens material	Fibre type	Wavelength	Measured NA	Trapping application	Reference
Refractive microprism	Two-photon lithography	Polymer	4 SMF bundle	1070 nm	1.15 water (theor.)	Red blood/ tumour cells	ref. ^51^
Diffractive meta-lens	fs direct laser writing	Polymer	1 SMF + MMF spliced	660 nm	0.882 water	2 µm beads/ E. coli	This work
Refractive ball lens	Glue	SiO_2_	1 SMF + MMF spliced	980 nm	0.875 water	0.2 µm beads/yeast cells	ref. ^52,53^
Digital holography	Spatial light modulator	–	1 MMF	1064 nm	>0.8 water	1.5 µm beads	ref. ^14^
Diffractive meta-lens	fs direct laser writing	Polymer	2 SMFs + spacer printed	808 nm	0.7 water	1 µm/ 0.5 µm beads	ref. ^22^
Plasmonic nanorods	Focused ion beam milling	Au	1 PCF	1550 nm	0.37 air	–	ref. ^18^
Refractive GRIN lens	Stack & draw + glue	SiO_2_	1 SMF + spacer glued	976 nm	0.16 air	2 µm beads (on the surface)	ref. ^54^
Refractive microlens	Laser exposure	Polymer	1 SMF + MMF spliced	980 nm	?	8 µm beads/ yeast cells (on the surface)	ref. ^55^
Diffractive Fresnel plate	Focused ion beam milling	SiO_2_	1 SMF + MMF spliced	980 nm	?	8 µm beads/ yeast cells (on the surface)	ref. ^17^
Diffractive Fresnel plate	UV-nanoimprint lithography	Polymer	1 SMF	660 nm	?	–	ref. ^30^
Refractive microaxicon	HF chemical etching	SiO_2_	1 SMF	633 nm	?	–	ref. ^19^

Our work is highlighted in grey

## Discussion

In this work, we have demonstrated that combining a nanoprinted ultra-flat meta-lens with an end-face modified step-index SMF leads to the realization of a novel type of strongly focusing meta-fibre device exhibiting a record-high numerical aperture of up to 0.9 and diffraction-limited spot sizes, allowing the optical trapping of microbeads and bacteria with a single SMF in combination with diffractive optics for the first time. The exceptionally high numerical aperture of the meta-lens enhanced fibre and its capability to optically trap objects with a single meta-fibre device enables one to envision applications in areas that are currently inaccessible by state-of-the-art devices. In contrast to electron beam lithography, 3D nanoprinting allows for applications in a wide range of fields due to the fast and cost-effective implementation of biocompatible devices that can be straightforwardly adapted to specific requirements. Potential fields of application include fibre-optic-based confocal microscopy, beam collimation for waveguide coupling, fibre lasers, fibre-based trapping and manipulation in quantum technology, bioanalytics, and life sciences. With respect to the latter, the introduced meta-fibre device may represent a first feasible pathway towards in vivo applications that include strong light focusing operation in difficult-to-access areas, e.g., high-resolution scanning-based tissue imaging.

Further improvements of the UNM design in terms of NA can be achieved by using narrowband laser sources and by more precise cleaving of the MMF or better characterization of the extended beam, both of which reduce the overcompensation of the wavefront curvature (Fig. 3e, g). The spatial resolution of the DLW system used (Δ*x* = 300 nm) limits the meta-lens phase profile discretization, which can be improved by recent advancements in DLW technology. As reported in ref. ^33^, stimulated-emission-depletion^34^ yields resolutions down to Δ*x* = 9 nm, allowing for further enhancement of the NA, as suggested by Fig. 2b. Increasing the refractive index of the liquid medium represents another way to increase the NA (e.g., by using immersion oil^35^), while here, we chose water to maintain biocompatibility and applicability. Note that dimethyl sulfoxide is an interesting candidate that is widely used in biology, with a refractive index as high as that of fused silica^36^. In addition, the concepts demonstrated in refs. ^37–39^ provide a new perspective for further increasing the transmission efficiency and controlling the chromatic aberration of meta-fibres. Furthermore, we envision the use of our UNM as a fibre-optic endoscope, as demonstrated in ref. ^40^, and consider the use of novel materials^41^.

## Materials and methods

### Experimental design and sample preparation

The fibre facet was functionalized by a silanization process prior to DLW, leading to better chemical and mechanical stability of the UNM^42^ and improved adhesion between the SiO_2_ and a polymeric microstructure^43^. To remove organic residue, the fibre tip was cleaned before by successively sonicating in acetone, isopropyl alcohol, and distilled water (15 min each) and drying with nitrogen. Subsequently, oxygen plasma activation was performed for 1 min (150 W) followed by overnight immersion in 1% 3-(trimethoxysilyl)propyl methacrylate (Sigma-Aldrich 440159) dissolved in ethanol and drying with nitrogen. The on-fibre UNM was fabricated by using a commercial DLW system with a 63× objective (NA = 1.4) to polymerize the negative photosensitive IP-Dip resist (Nanoscribe GmbH). For high-resolution 3D nanoprinting, we chose the commercial IP-Dip photoresist that offers a high spatial resolution down to 300 nm. Alternatively, IP-L photoresist with stronger mechanical stability but a reduced spatial resolution of 500 nm can also be used for creating 3D nanopillar-based birefringent metasurfaces^44^, which can potentially extend the meta-fibre capabilities for controlling the additional degrees of freedom of light, including amplitude, polarization, and orbital angular momentum. A 5 µm-thick base layer was first printed to promote the strong mechanical connection of the lens and fibre surface, followed by the actual printing of the UNM (50 nm hatching, 200 nm slicing, approximate printing time 1 h). After printing, the on-fibre UNM was developed in a propylene glycol monomethyl ether acetate (PGMEA, Sigma-Aldrich 484431) bath for 20 min followed by a 2 min Novec^TM^ (Sigma-Aldrich SHH0002) rinse. The final sample was rinsed with isopropyl alcohol before measurement.

### Focal scan measurement

The focus characterization relies on in-coupling a CW laser diode (*λ*_0_ = 660 nm, Δ*λ*_0_ = 13 nm, Thorlabs L660P120) to the water-immersed sample and imaging the beam from the front via a water-dipping objective (Nikon 60×, NA = 1.0) and a subsequent achromatic tube lens (*f* = 300 mm) onto a CMOS camera (Basler acA640-750uc, pixel size 4.8 µm). The focal position of the objective is axially scanned with a piezo-driven objective collar (Mad City Labs Nano-F100) in steps of size Δ*z* = 100 nm. At each position, micrographs are taken for three different exposure times (0.1 ms, 1 ms, and 10 ms), which are combined afterwards in a postprocessing routine to create HDR images with a noise floor smaller than 10^−5^.

### Optical trapping procedure

For the optical trapping experiments, light from a CW laser diode (*λ*_0_ = 660 nm (Thorlabs L660P120), shown in Fig. 4a) is coupled into the meta-fibre device, leading to an output power of *P*_out_ = 37 mW after the fibre meta-lens combination. The functionalized fibre tip is placed inside a water-filled chamber containing the particle solution (2 µm silica beads, Micromod Inc., concentration *c* ∼ 5 · 10^6^ cm^−3^) consisting of two parallel microscope cover glasses spaced ~0.5 mm apart. The motion of a trapped bead is recorded from the side via a home-built microscope (50× Olympus dry objective in combination with an *f* = 200 mm tube lens, resulting in 55× magnification) using a high-speed camera (Basler piA640-210m, pixel size 7.4 µm). A fast frame rate (*f* = 1 kHz) and a very small exposure time (*τ* = 10 µs) are used to minimize motion blur. Homogeneous particle illumination was provided by a Koehler-type arrangement (wavelength of 455 nm, as shown in Fig. 4c) consisting of an LED coupled to a delivery MMF (NA = 0.22, the core diameter of 105 µm (Thorlabs FG105LCA)). The light emitted from the MMF is first collimated using an *f* = 6.24 mm lens followed by a beam de magnifying telescope (*f* = 150 mm and *f* = 8 mm), resulting in the final illumination with NA = 0.16 and a spot size of 150 µm.

### Trajectory determination

For evaluation of the trapped particle behaviour, the trap stiffness is determined from recordings at different power levels by introducing neutral density (ND) filters before the in-coupling side of the fibre. As shown in Fig. 4d, the trajectory of a trapped bead is presented, extracted by centring each frame at time *t* via a Fourier shift algorithm assuming mirror symmetry (see ref. ^13^ for details). This yields the displacement *x*_⊥,||_(*t*) in the directions perpendicular (*x*_⊥_ = *y*) and parallel (*x*_||_ = *z*) to the fibre axis and ultimately results in the complete track of a trapped particle on timescales of >1 min.

### Statistical analysis and details of the fitting routine

The calculated PSD presented in Fig. 5a is first compressed by binning via Welch’s method to reduce error from noise and correlation^45–47^. Afterwards, it is fitted to the power spectrum PSD(*f*) = 2*D*/(4π^2^(*f*_c_^2^ *+* *f*^2^)) *+* *ε*^2^ via a maximum likelihood estimate for statistically correlated data^46–48^ to retrieve the fit parameters, namely, corner frequency *f*_c_ (where the power spectrum drops to half) and the free particle diffusion coefficient *D*. In comparison, Fig. 5b illustrates the corresponding MSD calculated for the same particle displacement *x*_*⊥,||*_(*t*) and fitted using MSD(Δ*t*) = 2*D*/(2π*f*_c_) · (1 − exp(−2π*f*_c_Δ*t*)) *+* *ε*^2^ including weights to compensate for correlation^29,48–50^. The error, *ε*^2^, results from uncertainty in tracking the particle position and is a measure of the motion blur due to excessively long exposure times. Here, the exposure time was chosen to be very small (*τ* = 10 µs).

## Supplementary information

Supplementary Information

Exchange of 2µm silica beads inside the trap (sample 1, left is the meta-fiber)

Exchange of 2µm silica beads inside the trap (sample 2, left: meta-fiber)

Zoom of a 2µm silica bead trapped over >1min

Proof of trapping (bead) by moving the meta-fiber against the background

Proof of trapping (E. coli) by moving the meta-fiber against the background

Zoom of an E. coli bacterium trapped over 1min

## Data Availability

The data that support the plots within this article and other findings of this study are available from the corresponding author upon reasonable request.
