# Supplementary material for: Ultrahigh numerical aperture meta-fibre for flexible optical trapping
Source: Light Sci Appl. 2021 Mar 15;10:57. doi: 10.1038/s41377-021-00491-z (PMC7960731; doi:10.1038/s41377-021-00491-z)
Supplement: Supplementary file 1 — Supplementary Information [file 41377_2021_491_MOESM1_ESM.docx]

**Supplementary Information for**

**Ultrahigh numerical aperture meta-fibre for flexible optical trapping**

**Malte Plidschun^1,2^, Haoran Ren^3^, Jisoo Kim^1,2^, Ronny Förster^1^, Stefan A. Maier^3,4^ and Markus A. Schmidt^1,2,5*^**

^1^Leibniz Institute of Photonic Technology, 07745 Jena, Germany. ^2^Abbe Center of Photonics and Faculty of Physics, FSU Jena, 07745 Jena, Germany. ^3^Chair in Hybrid Nanosystems, Nanoinstitute Munich, LMU München, 80539 München, Germany. ^4^Department of Physics, Imperial College London, London SW7 2AZ, UK. ^5^Otto Schott Institute of Materials Research, FSU Jena, 07745 Jena, Germany. ***Corresponding author:** [markus.schmidt@leibniz-ipht.de](mailto:markus.schmidt@leibniz-ipht.de)

**Supplementary Results**

**1 Meta-lens design**

To create a focal point at axial distance *z=f*, an ideal spherical phase *ϕ* is required which is realized here via a hyperbolic function $\phi_{\mathrm{hyp}}\left( r,f \right)=-k\cdot\left( \sqrt{r^{2}+f^{2}}-f \right)$ (ref. 1,2), where *k* is the wave vector 2π*n*/*λ*_0_ in the medium of refractive index *n* (vacuum wavelength: *λ*_0_, radial coordinate: *r*). Note that in many situations a parabolic approximation $\phi_{\mathrm{par}}\left( r,f \right)\approx\phi_{\mathrm{hyp}}\left( 0,f \right)+\frac{\partial}{\partial r}\phi_{\mathrm{hyp}}\left( 0,f \right)\cdot r+\frac{\partial^{2}}{\partial r^{2}}\phi_{\mathrm{hyp}}\left( 0,f \right)\cdot r^{2}/2+\ldots=-kr^{2}/\left( 2f \right)\mathcal{+O}\left( r^{4} \right)$ is used in literature^3,4^, which leads to wave front aberration due to the approximated phase and is therefore not considered in this work. For the ultraflat meta-lens design used here, the phase profile *ϕ* is discretized in steps of 2π into Fresnel zones via *ϕ*_kin_*=*mod(*ϕ*,2π), the so called kinoform^4–7^. Fig. S1a compares the two different lens profiles (*ϕ*_hyp_ and *ϕ*_par_) as wells as the discretized hyperbolic kinoform *ϕ*_kin_. Note that the profiles are calculated for a focal length *f*_0.8_*=r*_max_/tan(asin(NA*=*0.8/*n*)) required in long-term stable optical trapping^8^. The radial coordinate *r=*0…*r*_max_ is thus directly correlated to the numerical aperture NA*=n*sin(atan(*r*/*f*_0.8_)).

**2 Discretization**

Based on the Nyquist-Shannon theorem, spatial discretization limits the resolvable increase of phase Δ*ϕ* between two neighboring pixels Δ*x* by |*ϕ*'|≈|Δ*ϕ*/Δ*x*|<π/Δ*x*. In case this limit is violated, higher diffraction orders emerge, which overall result from undersampling, i.e., aliasing. This effectively reduces the usable lens diameter 2*r* and related NA while the focal length *f* stays constant. Fig. S1b shows the incremental increase of phase functions *ϕ* introduced in Section 1, where the lowest resolution was assumed to be Δ*x*≳300 nm (defined by the Abbe criterion in the two-photon based optical 3D printing). Note that towards the boundaries of the lens (*r*→1) the hyperbolic phase profile *ϕ*_hyp_ exhibits less curvature than the parabolic profile *ϕ*_par_ and is therefore consequently used throughout this work. It has to be mentioned that the phase *ϕ*(*λ*_0_) strongly depends on the operation wavelength *λ*_0_, affecting the minimum resolution Δ*x*(*λ*_0_) that is needed to avoid aliasing. Here, the used wavelength of *λ*_0_*=*660 nm yields a minimum resolution of Δ*x*(*λ*_0_*=*660 nm)*=*350 nm ensuring alias-free operation and numerical apertures of NA*>*0.8.


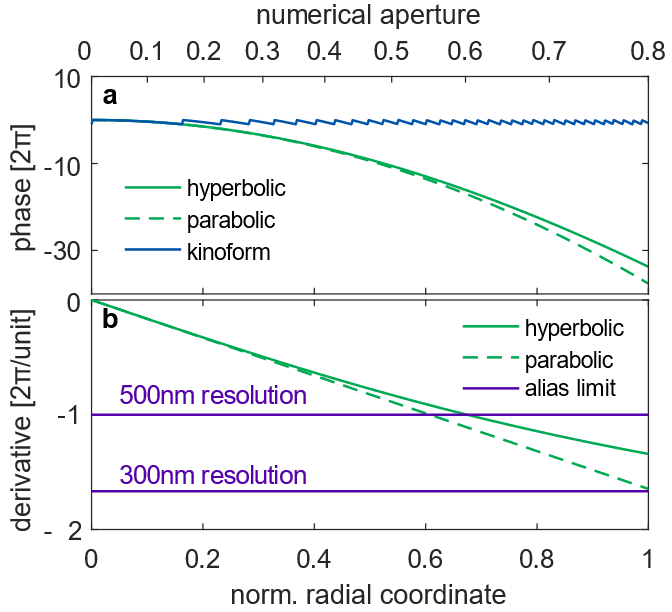


**Fig. S1 Design strategy of the ultrahigh-NA focusing fibre device.** **a** Comparison of hyperbolic and parabolic lens phase profiles (*ϕ*_hyp_ and *ϕ*_par_) commonly used, together with the discretized kinoform *ϕ*_kin_ (all calculated for NA*=*0.8). **b** Derivative of the phase profiles with respect to the radial coordinate. The horizontal lines indicate the minimum usable alias-free resolution, and the intersections with the derivative of the respective phase profiles yield the radial coordinate (i.e., maximum effectively usable lens diameter fraction) at which rays start to diffract into higher orders. Note that due to smaller curvature at the boundary of the lens, *ϕ*_hyp_ is used throughout this work.

**3 Coherence analysis**

To efficiently make use of the entire lens cross-section and NA, constructive interference of all lens elements needs to be provided, which is limited through the available laser source bandwidth Δ*λ*_0_ and the resulting coherence length *L*_coh_*=λ*_0_^2^/(*n*Δ*λ*_0_). The lens diameter 2*r* corresponds to the number *N*_Fres_*=ϕ*(*r*,*f*)/(2π) of so-called Fresnel zones, where each ring contains phases *ϕ*ϵ[0,2π) visible on the *y*-axis of Fig. S1a (*N*_Fres_≈40 for 2*r=*90 µm) and in Fig. S2. To achieve highest lens performance and efficiency, the constructive interference of all Fresnel rings is required, demanding *L*_coh_*>λ*_0_*N*_Fres_. The coherence limit for a laser diode with broad bandwidth Δ*λ*_0_ is the main limiting factor for ultrahigh NAs, while in contrast, resolution Δ*x* and aliasing are limiting factors for lower, still high NAs. Here we designed two samples with different parameters: (i) sample 1 with spatial resolution Δ*x*_1_*=*330 nm and focal length *f*_1_*=*55 µm, corresponding to NA*=*0.84; (ii) sample 2 with Δ*x*_2_*=*300 nm and *f*_2_*=*50 µm, corresponding to NA*=*0.88. The lens diameter was chosen to be 2*r=*90 µm for both samples.

**4 Curved wave front compensation**

After leaving the optical fibre’s aperture, the output profile of conventional step-index fibres diverges with curved wave fronts of radius *R*(*z*)*=z*⋅(1+*z*_R_^2^/*z*^2^)≈*z* (*z*_R_: Rayleigh length) for propagation distances *z*≫*z*_R_∼10…30 µm, representing typical Rayleigh lengths of commercially available step-index fibres in the visible. Hence, the fibre acts as a negative defocusing lens with *f=*-*R*(*z*) and exhibits its own spherical phase $\phi_{\mathrm{fib}}\left( r,z \right)=k\cdot\left( \sqrt{r^{2}+z^{2}}-z \right)$, which reduces the overall phase *ϕ*_tot_(*r*,*f*,*L*)*=ϕ*_hyp_(*r*,*f*)*+ϕ*_fib_(*r*,*L*) of the combined system (meta-lens on expansion fibre of length *L*). To improve focusing performance we included a correction term to compensate for spherical aberration of the fibre beam, which is a key advantage of using meta-lenses since any phase anomalies can be straightforwardly corrected.

Fig. S2a–c illustrates a simulated lens phase profile *ϕ*_hyp_(2*r=*90 µm,*f*_2_*=*50 µm) (sample 2, NA*=*0.88) without wave front correction illuminated by the output mode. The beam expansion section is assumed to be *L*_2_*=*750 µm which is sufficiently long so that the beam fills out the lens aperture. The inset in the figure refers to the radial focal plane, while the dashed line indicates the width of a fitted Airy function (scale bar: 500 nm). The solid curves in Fig. S2b, c show the intensity distribution along the symmetry axes of the focus, while the dashed lines refer to fits according to diffraction theory (radial intensity: *I*(*r*)∼jinc^2^(2π*r*/*λ*_0_⋅NA), axial intensity: $I\left( z \right)\sim\mathrm{sinc}^{2} \left( \left( z-f \right)/\lambda_{0}\cdot\left( n-\sqrt{n^{2}-\mathrm{NA}^{2}} \right) \right)$) to retrieve the NA^9^. Here, jinc(*r*)*=*2*J*_1_(*r*)/*r* is defined as the Airy function including the Bessel function of first kind *J*_1_ and sinc(*z*) as sin(*z*)/*z*. Dotted lines represent the full-width-half-maximum (FWHM) of the fits. Note that strong spherical aberration from an uncorrected fibre wave front curvature reduces the NA by ~0.1 to *<*0.8 via shifting the focus in axial focal position by about Δ*f*∼5 µm, excluding this configuration from being useful for optical trapping.

In comparison, Fig. S2d–f shows the simulated focus including the wave front compensation by *ϕ*_tot_(*r*,*f*,*L*)-*ϕ*_fib_(*r*,*L*_min_*=*700 µm), reaching very high numerical aperture values of up to NA*=*0.88. Note that due to experimental cutting precision a minimum length *L*_min_*=*700 µm of the expansion section is assumed. This leads to an overcompensated fibre aberration which in general has less severe impact on lens performance than undercompensation and is also visible in measurements.


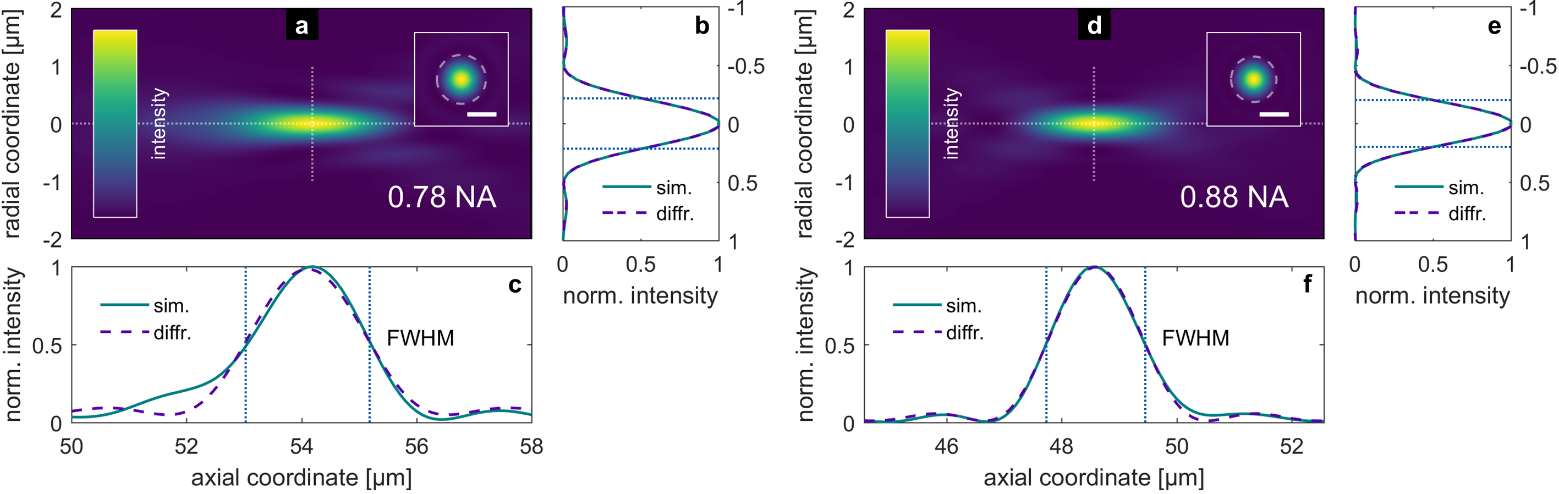


**Fig. S2 Simulated performance of the ultrahigh-NA focusing fibre device.** **a** and **d** Simulated intensity distributions of the meta-lens focus with uncompensated and compensated curvature. Insets refer to the distributions in the radial focal planes, with the dashed white lines indicating the widths of the fitted respective Airy functions (scale bars 500 nm). The corresponding intensity distributions along the symmetry axes (**b** and **e** radial direction, **c** and **f** axial direction) through the focal plane are additionally shown (solid lines) including fits (dashed) and full-width-half-maximum (FWHM, dotted). Note that the uncorrected case (**a** and **c**) leads to strong spherical aberration reducing the NA by ~0.1.

**5 Experimental error analysis and retrieval of the NA of the on-fibre UNM**

1. High-precision characterization of the lateral resolution: We calibrated the magnification of the optical microscope used for the meta-lens focus characterization by recording a reference image of the USAF resolution target. Based on the retrieval of this magnification we then evaluated the NA by fitting the experimentally recorded Airy disc of the measured on-fibre meta-lens focus to the corresponding equation stated in the manuscript.


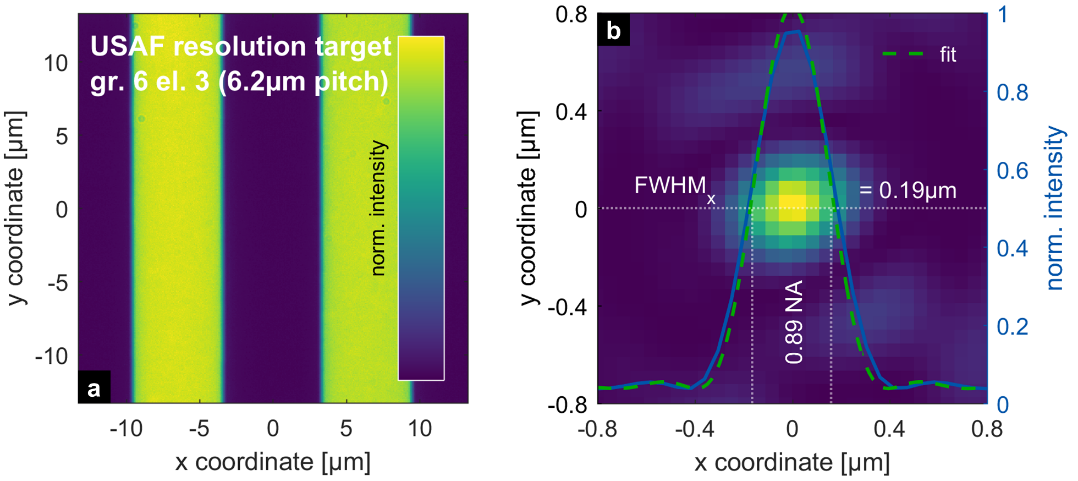


**Fig. S3 High-precision characterization of the lateral resolution.** **a** Calibration of the magnification of the optical microscope used for the characterization of the meta-lens focus via the USAF resolution target. **b** Recorded Airy disc of the measured on-fibre meta-lens focus and fit to the corresponding equation stated in the manuscript. FWHM*_x_* denoted the lateral full width half maximum and reveals a numerical aperture of 0.89.

The retrieved NA of 0.89 in lateral direction is in an excellent agreement to the previously stated value and is very consistent with the NA in the axial direction (see point 2 below). It deviates only slightly from this value due to realistic experimental errors.

1. High-precision characterization of the axial resolution: We carefully checked the LabVIEW code used for axially scanning the focus of the fibre-integrated meta-lens. We have made the measured raw data and the LabVIEW code for controlling the piezo scanning drive publicly available upon request. The resolution of the used piezo drive was set to 100 nm along the axial direction. According to the manufacturer, the precision lies in the order of sub-nm which corresponds to an error of <1% for the axial FWHM of the measured focus. Additionally, based on the stack of recorded images, the combination of lateral and radial direction together is used for the full three-dimensional retrieval of the NA of the on-fibre meta-lens (Fig. S4).

We performed a detailed error analysis and calculated that for an error of 5% the numerical aperture corresponds to NA=0.86 which denotes a deviation of 2% to our value. On the other hand, in order to significantly deviate from the retrieved value of NA=0.88 (e.g. NA<0.8), the error of the piezo scanning drive would have to be >23%, which would result in a completely incorrect operation that we did not observe in the measurement.


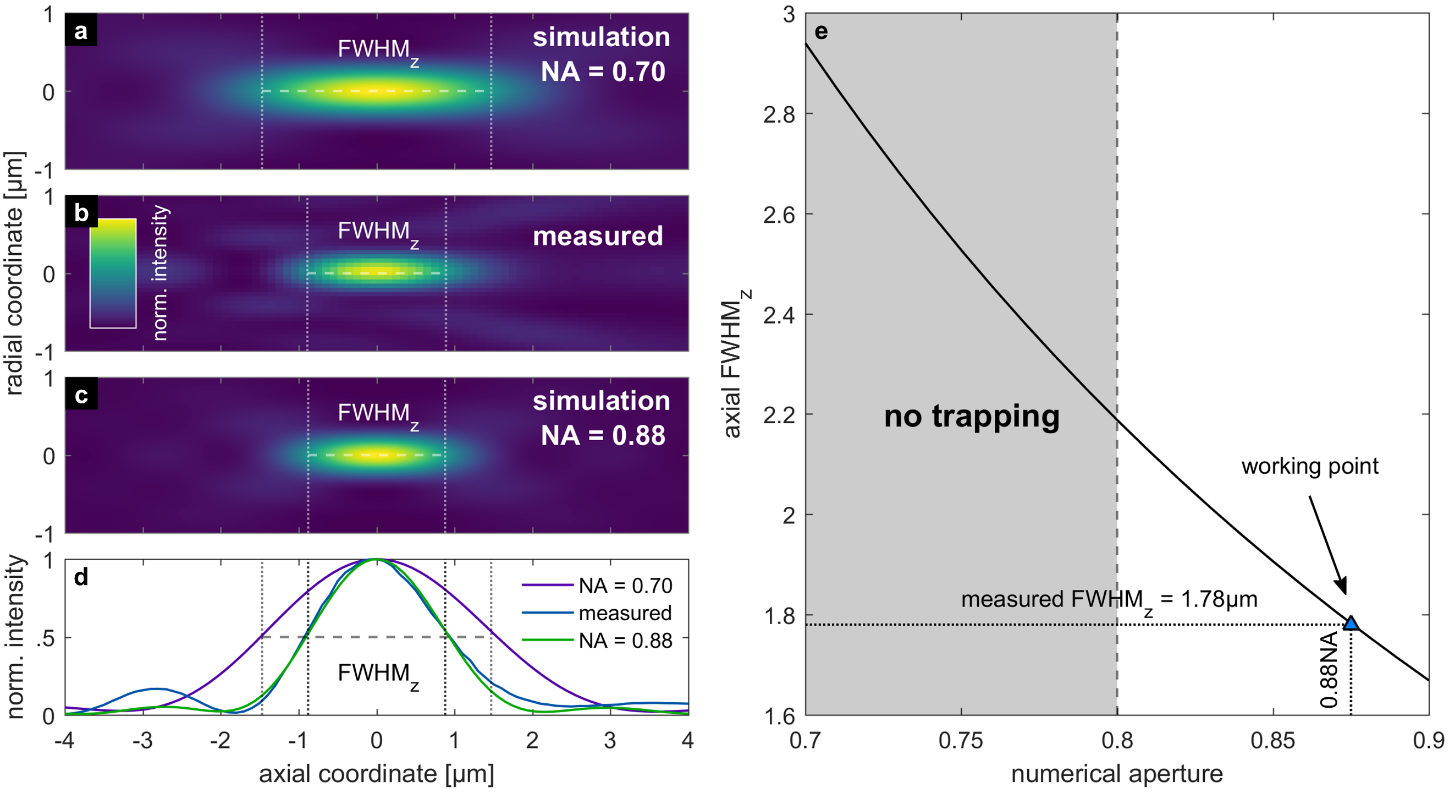


**Fig. S4 Comparison of diffraction-limited high-NA foci and relation to single-beam optical trapping.** **a** Simulated focus for a numerical aperture of 0.7. **b** Measured on-fibre meta-lens focus. **c** Simulated focus for a numerical aperture of 0.88. **d** Comparison of axial profiles along the horizontal dashed lines in **a**–**c**. The measured profile of **b** shows an excellent agreement with the simulated case of **c**. FWHM*_z_* denotes the axial full width half maximum. **e** Axial FWHM for different NAs. The measured value of 1.78 µm reveals a numerical aperture of 0.88.

1. Comparison of diffraction-limited high-NA foci: In Fig. S4 we have compared the experimental results of our measured on-fibre meta-lens focus (Fig. S4b) with the simulation results of diffraction-limited foci with two different NAs (0.7, Fig. S4a and 0.88, Fig. S4c). An excellent agreement between our measurement and simulation results on the axial resolution of the high NA of 0.88 (Fig. S4d) has been achieved, which further consolidates the high NA (0.88) of our 3D printed meta-lens (Fig. S4e).
2. Stable single-beam optical trapping for >1min: Optical trapping with a single-beam requires at least an NA of 0.8 for stable confinement >1h at a power of 1mW (see refs. [8,10]). The lowest power which was used for optical trapping in our experiment here was 3.7mW (OD1.0 density filter applied to the original power of 37mW) and the particles were stably trapped for minutes. The Kramer’s time for stable optical trapping with a single beam scales super-exponentially with the NA (see ref. [10]) revealing an NA of at least NA>0.78 for the observed stable optical trapping with a single on-fibre meta-lens on timescales >100s.

**Supplementary Figures**


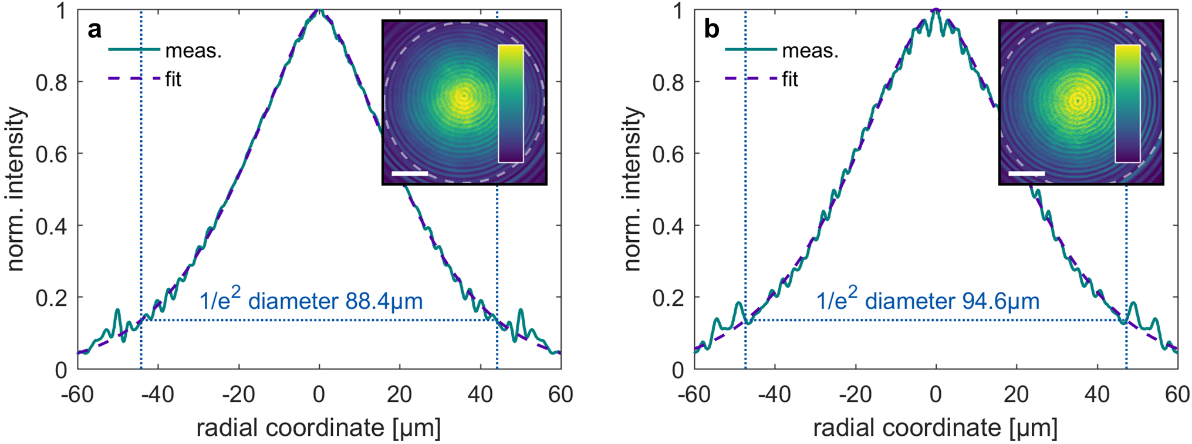


**Fig. S5 Optical properties of the functionalized fibre.** Measured intensity distribution of the fundamental mode at the end of the beam expansion section (**a** sample 1; **b** sample 2). The solid lines result from azimuthal averaging of the 2D intensity distribution shown in the insets (dashed circle: fitted mode diameter, scale bar: 20 µm). The purple dashed lines refer to fitting with a sub-Gaussian profile to determine the mode diameter (taken as 1/e^2^ value of the intensity). The colour in the inset ranges linearly from zero (dark blue) to unity (yellow). The ring-type structure of the beam visible in the insets results from interference with reflections from the core-cladding boundary of the MMF.


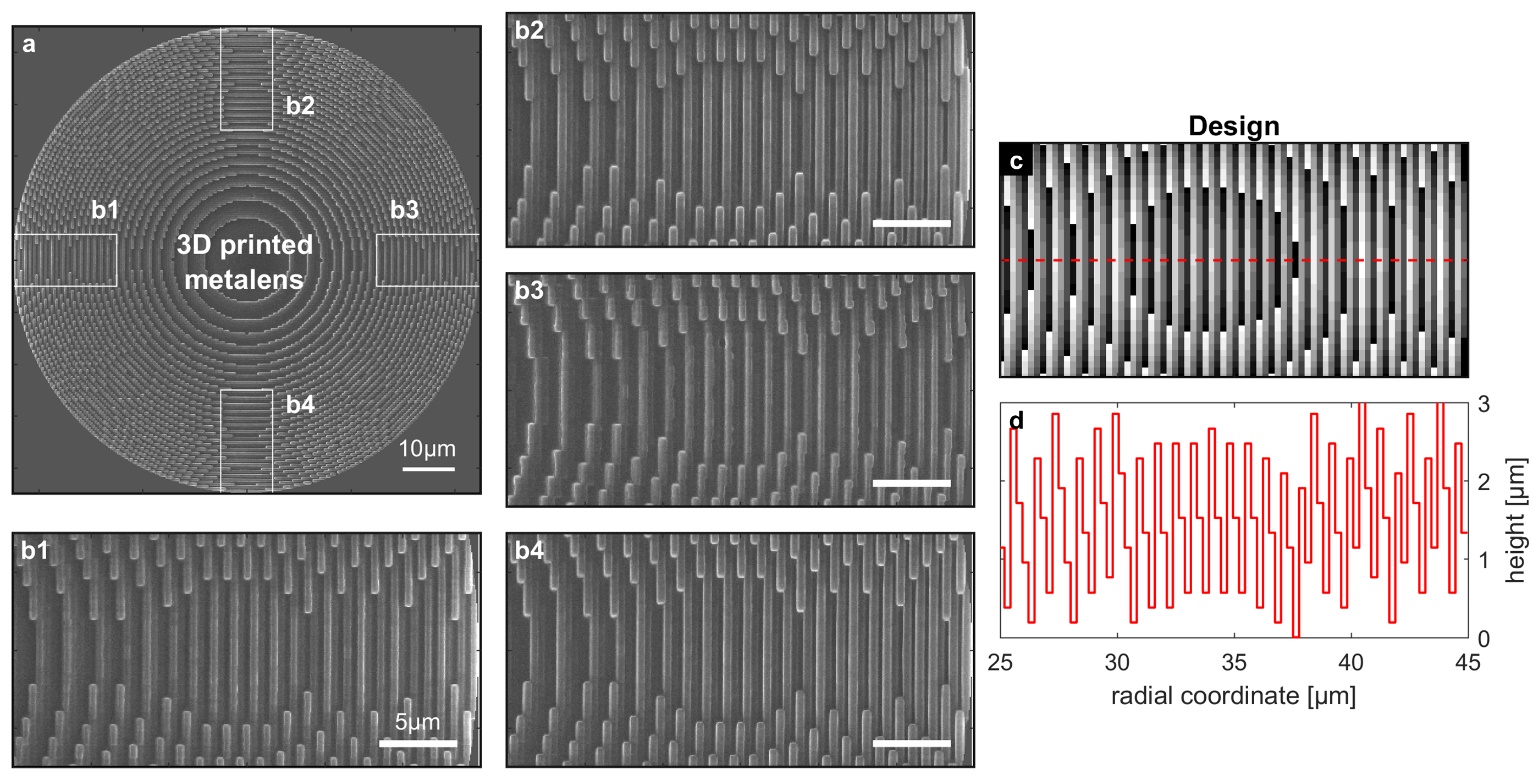


**Fig. S6 Close-up scanning electron microscope (SEM) images of a 3D nanoprinted meta-lens with 300 nm lateral resolution and comparison to the design.** **a** Overview of one selected fabricated meta-lens. **b1**–**b4** Close-up micrographs of the regions marked in **a**. **c** Corresponding design (i.e., distribution of the height of the elements) showing an excellent agreement with the printed regions of **b1**–**b4**. **d** Cut along the red dashed line in **c**. Note that the axes are not to scale, thus only representing the general concept of the discretization of the profile which is not to be mistaken with the sampling of a Fresnel lens (see main text).


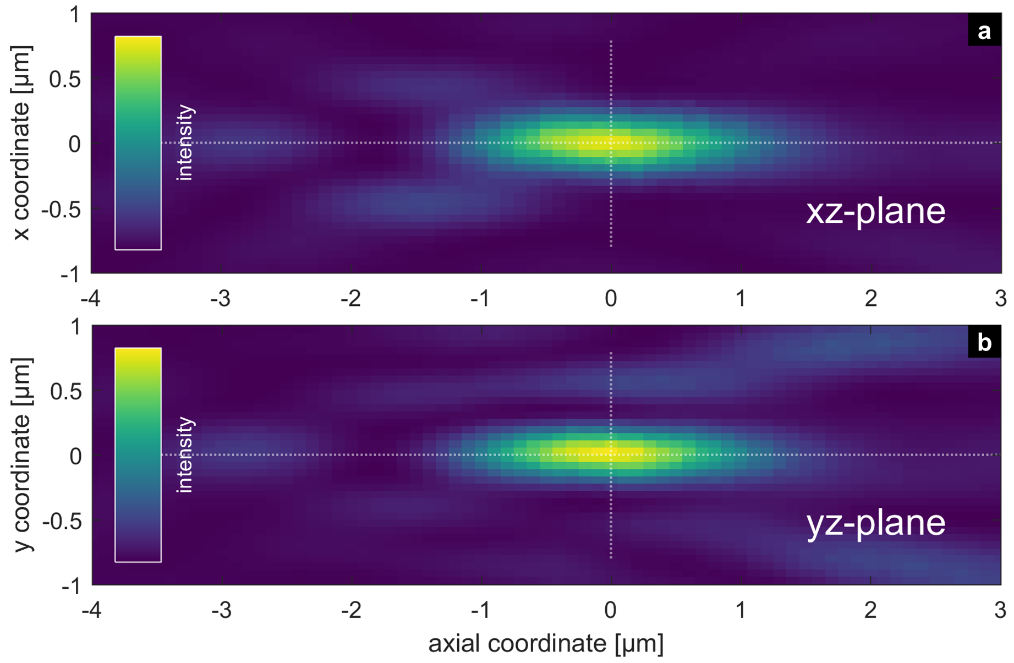


**Fig. S7 Cut planes through the measured focus of the on-fibre UNM in water (a *xz*-plane, b *yz*-plane).** The focal planes are in excellent agreement with the azimuthal average presented in Fig. 3 of the main text of the manuscript and show no signs of distortion into either of the directions.

**References**

1. Khorasaninejad, M. *et al*. Metalenses at visible wavelengths: diffraction-limited focusing and subwavelength resolution imaging. *Science* **352**, 1190-1194 (2016).
2. Yang, J. Y. *et al*. Photonic crystal fiber metalens. *Nanophotonics* **8**, 443-449 (2019).
3. Golan, L. *et al*. Design and characteristics of holographic neural photo-stimulation systems. *Journal of Neural Engineering* **6**, 066004 (2009).
4. Yamada, K. *et al*. Multilevel phase-type diffractive lenses in silica glass induced by filamentation of femtosecond laser pulses. *Optics Letters* **29**, 1846-1848 (2004).
5. Asadollahbaik, A. *et al*. Highly efficient dual-fiber optical trapping with 3D printed diffractive Fresnel lenses. *ACS Photonics* **7**, 88-97 (2020).
6. Neuman, K. C. & Block, S. M. Optical trapping. *Review of Scientific Instruments* **75**, 2787-2809 (2004).
7. Siemion, A. Terahertz diffractive optics—smart control over radiation. *Journal of Infrared, Millimeter, and Terahertz Waves* **40**, 477-499 (2019).
8. Kramers, H. A. Brownian motion in a field of force and the diffusion model of chemical reactions. *Physica* **7**, 284-304 (1940).
9. Amos, B., McConnell, G. & Wilson, T. Confocal microscopy. in Comprehensive Biophysics (ed Egelman, E.) (Amsterdam: Elsevier, 2012).
10. Leite, I. T. *et al*. Three-dimensional holographic optical manipulation through a high-numerical-aperture soft-glass multimode fibre. *Nature Photonics* **12**, 33-39 (2018).

**Supplementary Files**

Movie S1. Exchange of 2 µm silica beads inside the trap (sample 1, left is the meta-fibre).

Movie S2. Exchange of 2 µm silica beads inside the trap (sample 2, left: meta-fibre).

Movie S3. Zoom of a 2 µm silica bead trapped over *>*1min.

Movie S4. Proof of trapping (bead) by moving the meta-fibre against the background.

Movie S5. Proof of trapping (*E. coli*) by moving the meta-fibre against the background.

Movie S6. Zoom of an *E. coli* bacterium trapped over 1min.
